# Impact of transient currents caused by alternating drain stress in oxide semiconductors

**DOI:** 10.1038/s41598-017-10285-2

**Published:** 2017-08-29

**Authors:** Hyeon-Jun Lee, Sung Haeng Cho, Katsumi Abe, Myoung-Jae Lee, Minkyung Jung

**Affiliations:** 1 Intelligent Devices & Systems Research Group, Institute of Convergence, DGIST, Daegu, 42988 Korea; 2 Global Center for Bio-Convergence Spin System, DGIST, Daegu, 42988 Korea; 3 Realistic Display Research Group, ETRI, Daejeon, 34129 Korea; 4 Silvaco Japan Co., Ltd., Nakagyo-ku, Kyoto, 604-8152 Japan

## Abstract

Reliability issues associated with driving metal-oxide semiconductor thin film transistors (TFTs), which may arise from various sequential drain/gate pulse voltage stresses and/or certain environmental parameters, have not received much attention due to the competing desire to characterise the shift in the transistor characteristics caused by gate charging. In this paper, we report on the reliability of these devices under AC bias stress conditions because this is one of the major sources of failure. In our analysis, we investigate the effects of the driving frequency, pulse shape, strength of the applied electric field, and channel current, and the results are compared with those from a general reliability test in which the devices were subjected to negative/positive bias, temperature, and illumination stresses, which are known to cause the most stress to oxide semiconductor TFTs. We also report on the key factors that affect the sub-gap defect states, and suggest a possible origin of the current degradation observed with an AC drive. Circuit designers should apply a similar discovery and analysis method to ensure the reliable design of integrated circuits with oxide semiconductor devices, such as the gate driver circuits used in display devices.

## Introduction

From the time since Nomura *et al*. proposed^1, 2^ metal-oxide semiconductor thin film transistors as replacements for silicon-based devices in active matrix displays a decade ago, numerous studies have been conducted to investigate a variety of applications, as well as to understand the nature of the defects in these devices^3–5^. Oxide semiconductors have often been proposed for high performance applications, and have also been found to improve device reliability in various environments^6, 7^. Recently, faster drive speeds have been required of these devices, and Tin (Sn) doped oxide semiconductors have been suggested as suitable candidates for this purpose^8^. However, the high speed and high voltage drives that enable better performance in oxide semiconductors also cause “device degradation” due to charge trapping on the interface^9^, which forms defect states by self-heating^10^ or hot carrier^11^ in the devices. In particular, the degree of degradation in an oxide semiconductor is likely to decrease as the drive power is reduced because low voltage drives cause fewer hot carrier defects. Although there have been reports detailing the influence of gate^12^ and/or drain^13^ voltage stress dependence (applied direct current (DC)) and on the influence of alternating stress signals^14^, our understanding of the stresses induced by alternating high voltage and periodic pulses on the drain side remains incomplete because the drain voltage and frame rate (frequency) affect both the electrical properties and performance of oxide semiconductors. Thus, a complete understanding of the effects of the hot carriers and frame rates caused by alternating current drives on the electrical properties of amorphous oxide semiconductors is important to ensure the success of future electronics applications that leverage this technology.

In this paper, we investigate the impacts of hot electrons and drive frequencies in oxide semiconductors without a gate DC bias. In our testing, we employed amorphous indium gallium zinc oxide (a-IGZO) as the channel material for n-type oxide transistors fabricated on an insulating silicon oxide/silicon substrate. To detect a reduction, or degradation, in the current, drain current-gate voltage (I_d_ − V_g_) measurements were conducted in the presence of alternating stress signals on the drain side. Note that this test is different than the negative bias temperature illumination stress test (NBTIS), which is only applied to the gate side. Further characterization of the TFT revealed that the degradation was strongly dependent on the rise/fall time, and changes in the band gap states close to the drain side. More importantly, the results of this investigation show that the current degradation is likely to accelerate drastically when the oxide devices are subjected to high frame rates. However, after simulating the electric field concentration and observing the transient properties induced by alternating pulse signals, we found that this situation could be improved by controlling either the drain leakage current or the rise/fall time of the pulse signals. The transient properties caused by hot electrons were characterised using a device simulation.

## Results

### Current degradation while being driven

**Figure 1:**
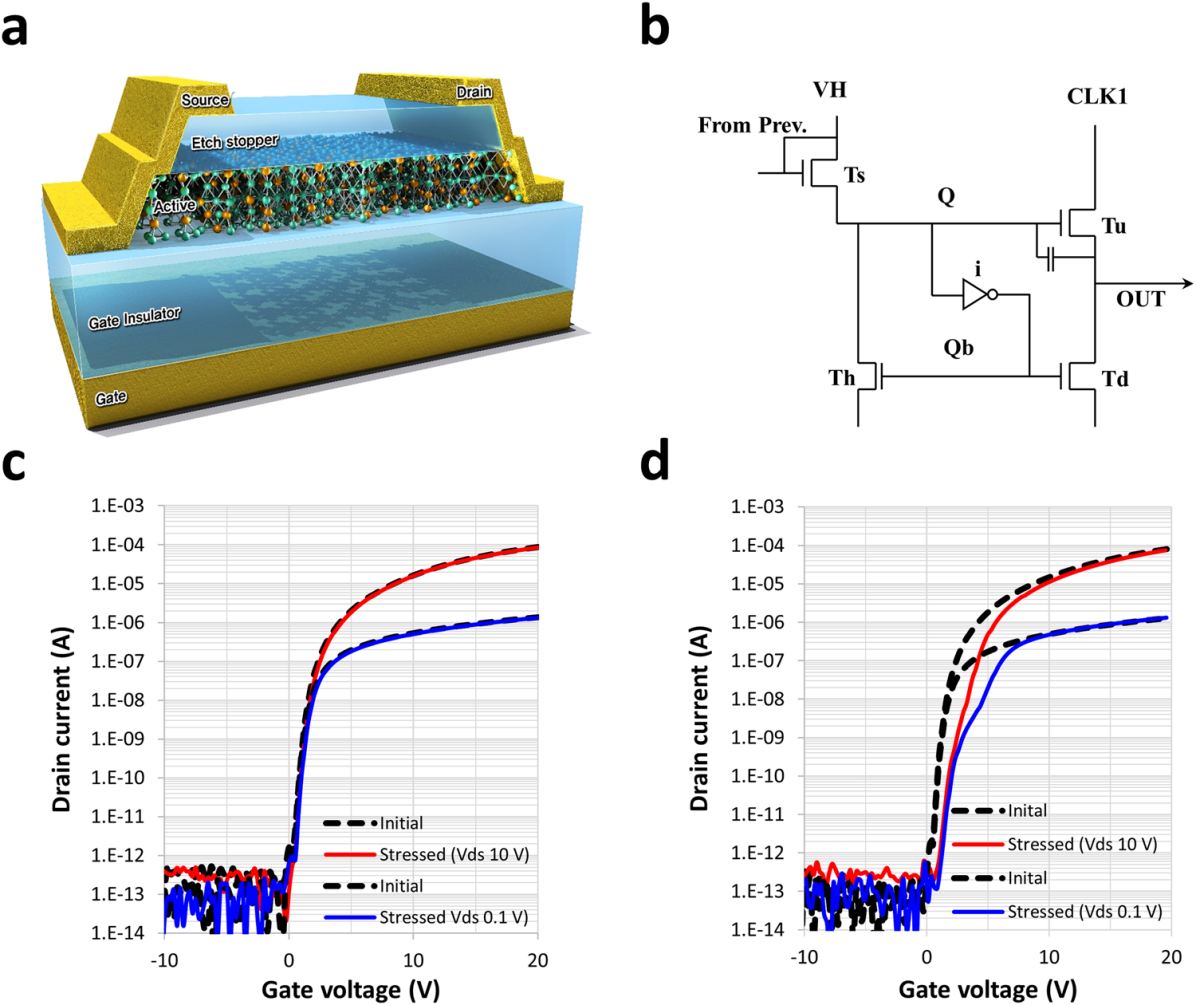
Oxide semiconductor TFT. (**a**) Cross sectional schematic of an a-IGZO (active layer) gate insulator and metal gate. (**b**) Schematic showing a unit stage of a conventional integrated gate drive circuit used in display devices^15^. (**c**) I_d_ − V_g_ characteristic with DC voltage stress applied, and (**d**) with AC voltage stress.

To select a line of pixels for image writing in a display device, a series of high voltage pulses is applied sequentially to the gate lines. Figure 1a and b show a cross sectional image of an oxide TFT and a schematic diagram of the unit stage of a conventional gate driver circuit used in display devices^15^. The display device, by way of the gate drive circuits, normally adapts a clock-driven sequence in which a clock line is connected to the pull-up TFT (Tu), as shown in Fig. 1b. When a signal is introduced from one of the previous stages by way of the Ts, the stage is set to be activated by the output pulse via the “Q-node high”. The Q-node is set to a higher voltage than the input signal from V_start_ via the boost-up process. The gate of the Tu and the source/drain of the Ts/Th will be affected by the high voltage pulse on the Q-node. The high voltage on the Q-node results in a variety of symptoms that can be observed using the well-known reliability test and observing the response to DC^10^ and pulsed^16^ gate bias stress as a function of time under temperature/illumination. The effect of applying a high voltage to the Q-node on the drain side, e.g., in the Ts or Th transistor in Fig. 1b, was investigated using two different type of bias stress, namely, DC and AC signal stress. Figure 1c shows the transport characteristics when DC voltage stress was applied to the active channel through the drain electrode. A DC voltage stress of 40 V was applied to the drain electrode while the gate and source were grounded. The I_d_ − V_g_ sweep characteristic after the stress was applied (86 ks) was similar to that of the initial sweep data. However, the transport properties drastically changed when the AC pulse signal was applied. The AC signal was a sequential pulse signal similar to that used for a commercial gate drive circuit, and was applied to the drain electrode, as shown in Fig. 1d. The threshold voltage experienced a small shift toward the right side, and the current at the threshold region dropped significantly for the drain voltages of 10 V and 0.1 V. A hump behaviour was observed on the plot representing the V_ds_ 0.1 V test with the AC signal, whereas there was neither a hump nor a shift under DC bias stress. To the best of our knowledge, the presence of this current degradation in oxide semiconductor TFTs has not been previously reported. During the testing, the drain voltage was fixed at 40 V and a frequency of 1 kHz was used.

**Figure 2:**
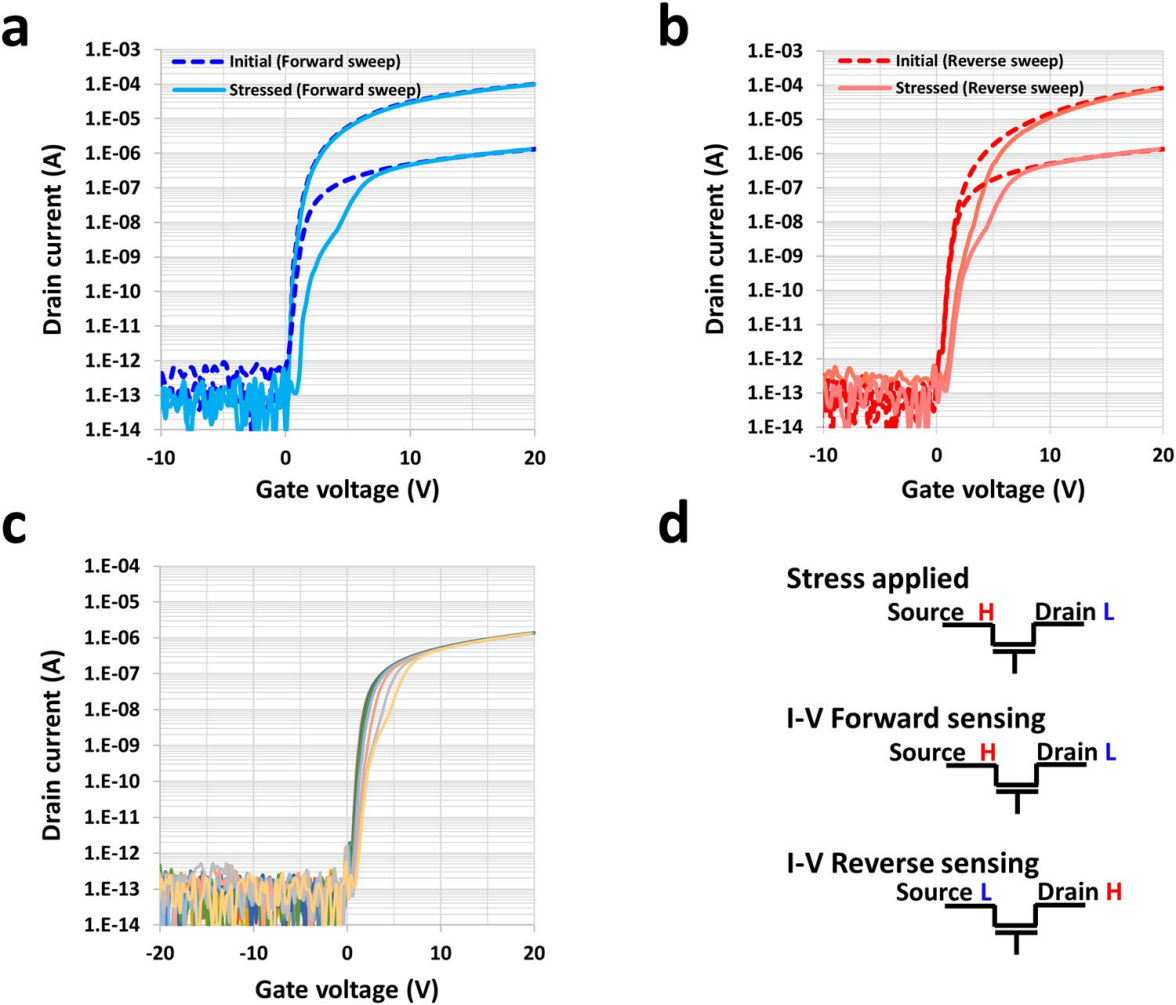
Device characteristics initially and after stress has been applied. (**a**) I_d_ − V_g_ forward characteristics for V_ds_ = 10 V and 0.1 V before and after drain stress was applied (V_ds_ = 10 V before the stress: blue dotted-line, V_ds_ = 0.1 V before the stress: red dotted-line, V_ds_ = 10 V after the stress: light blue line, and V_ds_ = 0.1 V after the stress: light red line), and (**b**) the reverse characteristics. (**c**) I_d_ − V_g_ characteristics at V_ds_ = 0.1 V as function of the stress duration up to 24 h. (86,000 s; bright origin line). The I_d_ − V_g_ sweeps were carried out at the 1; 10; 100; 350; 1,000; 3,500; 10,000; 35,000; and 86,000 s, and the graph covers from 0 to 86,000 s. (**d**) Notation of the stress applied and the I_d_ − V_g_ forward and reverse sweep for sensing.

Figure 2a and b show the changes in the transfer characteristic caused by the high voltage at the drain side (HVDS) (1 kHz frequency, duty cycle 1%, t_on_ = 1 μs, turn on voltage (V_ds_) = + 40 V, and turn off voltage = 0 V @ Vg = 0 V). In the V_ds_ = 0.1 V I_d_ − V_g_ sweep, a current drop and small positive shift were observed in both directions. The forward (V_ds_ sweep @ HVDS) and reverse (V_sd_ sweep @ HVDS) I_d_ − V_g_ measurements are shown in Fig. 2d. However, the asymmetric transfer characteristic for the V_ds_ 10 V I_d_ − V_g_ sweep was similar to that observed in Si based TFTs under DC stress^17^. This can be explained by noting that the high electric field near the drain region accelerates the electrons, thereby causing the weak oxygen bonds to be broken (which is related to oxygen deficiency). This results in the increase of tail sub-gap states in the band gaps of the host matrix. The current at low gate voltages (near the threshold) drastically decreases over the duration of the test up to 86 ks, and the drop at 86 ks is similar to the hump characteristics in the I_d_ − V_g_ measurement shown in Fig. 2c. In terms of the small positive shift, this originated from either the negative fixed charge at the interface between the gate insulator (GI) and active layer because of the high positive drain voltage or the generation of (increase in) the deeper (E − E_C_ < −0.4 eV) acceptor-like defects close to the conduction band^18^.

### Strong driving frequency dependence

**Figure 3:**
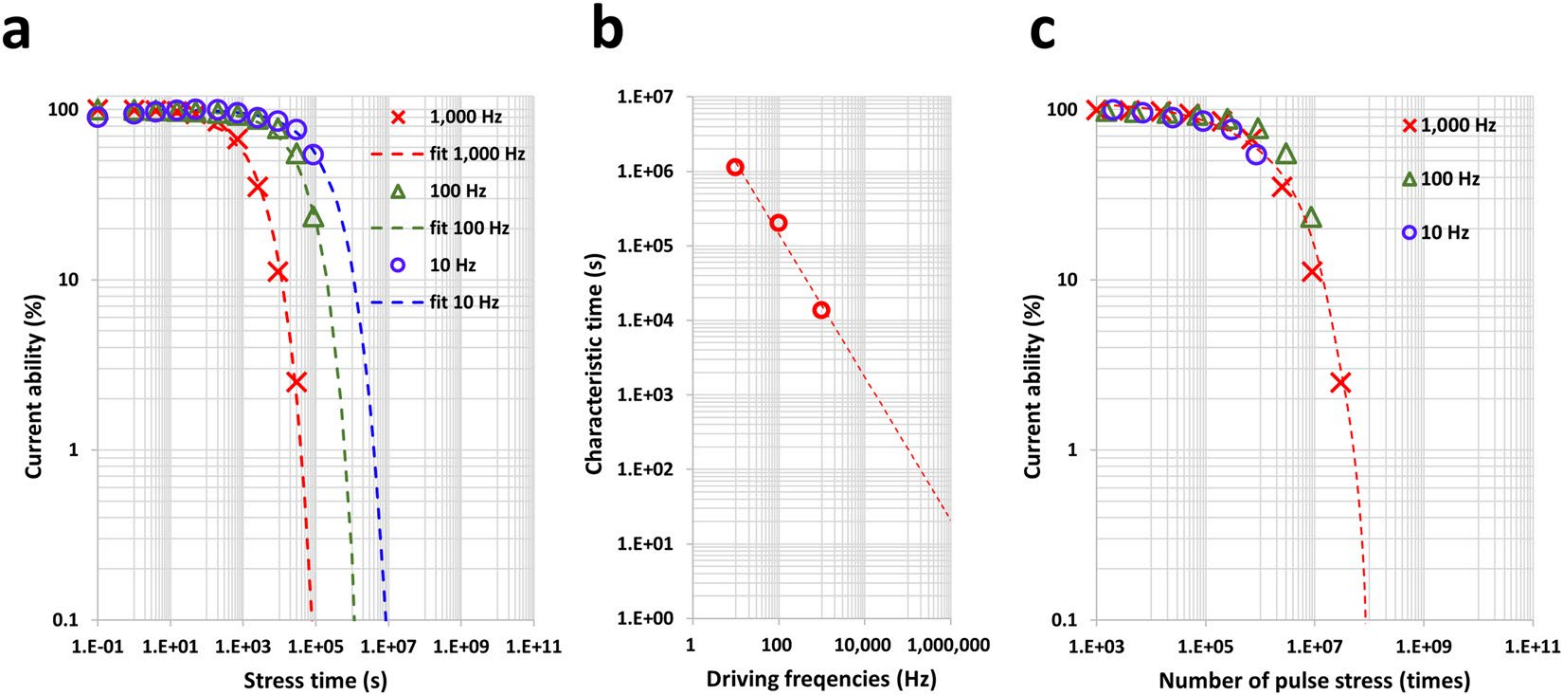
Current capability and characteristic time vs. the driving frequency. (**a**) Current (current @ V_g_ 5 V and V_ds_ 0.1 V) trend as a function of the HVDS time. Three different frequencies were applied: 10, 100, and 1,000 Hz. The degradation trends were fitted with a stretched exponential function. (**b**) The characteristic times τ that were extracted by the stretched exponential function shown in **a**, show the dependency on the frequency. (**c**) The current under HVDS conditions at various frequencies shows strong dependence on the number of pulse times.

Recently, many electronic devices have come to require high speed drives, and the clock rates have gradually increased from the traditional 60 Hz to 120, 144, 240, and even 480 Hz for displays. Figure 3a shows the current capability in the V_ON_ (V_ON_ is the voltage at the gate, which is 5 V) state as a function of the HVDS time with various driving frequencies. As the HVDS time increases, the current capability is drastically reduced. The shape of the corresponding curve is well represented by a stretched exponential function, which is often used as a phenomenological description of relaxation in disordered systems^19^. The equation for the stretched exponential curve used for fitting the data is as follows:

1 $${\rm{\Delta }}I(t)={I}_{0}\exp [-{(t/\tau )}^{\beta }],$$

where *I*_0_ is *ΔI*(*t*) at the initial current level normalised as a percentage and τ is the time constant and is referred to as the characteristic time^20^, which is strongly proportional to the driving frequency and is well matched to the power law (Fig. 3b). The equation describes the superposition of processes with a spread of time constants around an average value τ, the spread being quantified by the stretch parameter β < 1. The fitting curves are shown by the dashed line in Fig. 3a. The stretch parameter β was determined to be 0.55–0.6 (within the margin of error), and exhibited independent behaviour as the frequency changed. This means that the origin of the current drop (degradation) does to change with various driving frequencies because the stretch parameter indicates the rate of degradation in terms of the phenomenological behaviour^19^. In addition, no degradation was observed when high voltage was applied to the drain side without a periodic signal. Figure 3c shows the relationship between the current capability and the number of pulses, which were obtained based on the applied frequencies in the HVDS test. The current capability data for the three different frequencies exhibited an overlap along the same axis, and was strongly dependent on the number of pulses. Consequentially the frequency dependence of the HVDS test reflects the dependence on the number of applied pulse signals.

### Supplying hot electrons via the leakage current

**Figure 4:**
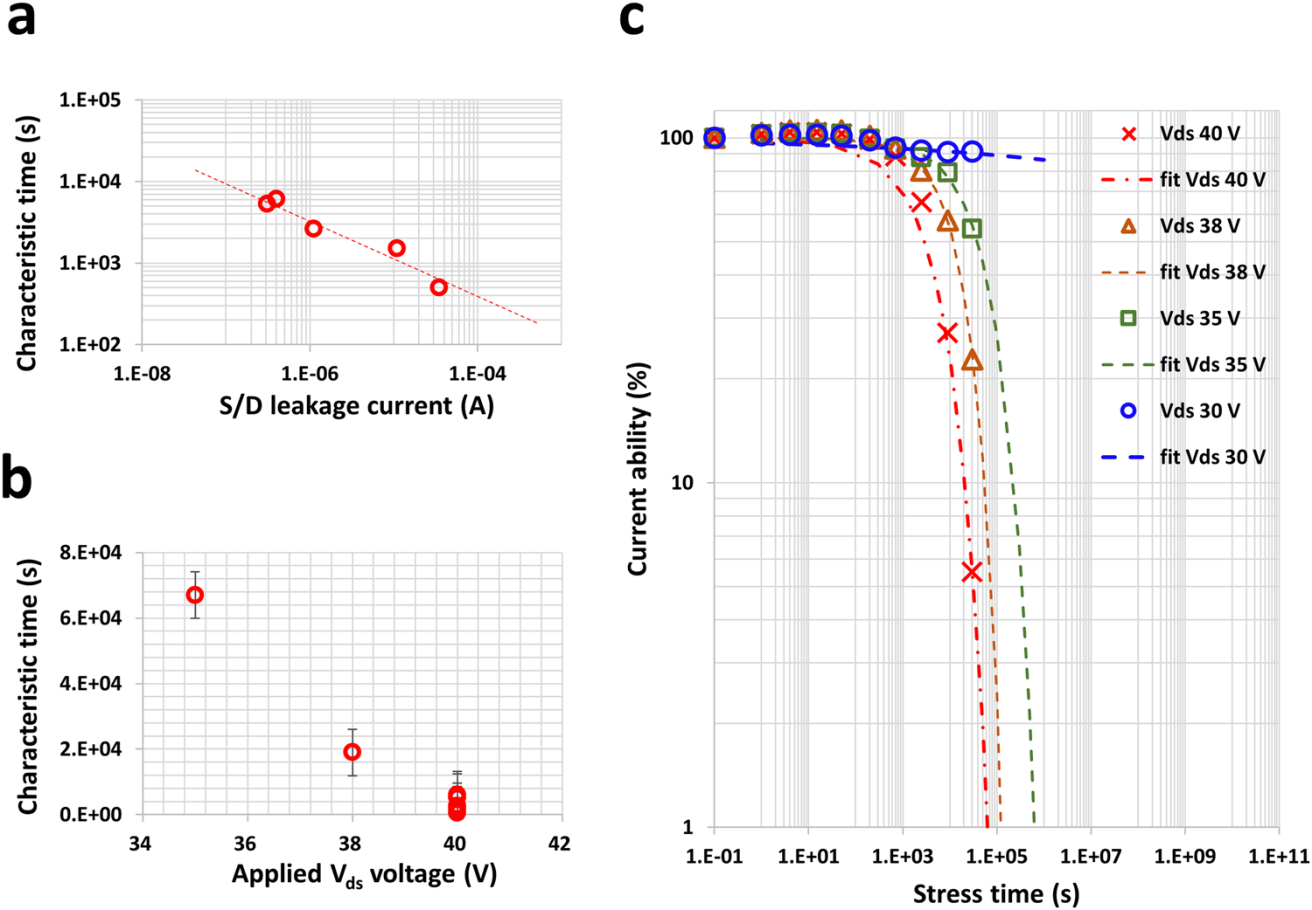
Device degradation characteristics vs. the applied voltage. (**a**) Extracted characteristic times show the dependence of the leakage current between source and drain when the gate is off. (**b**) The degradation accelerates during high voltage stress. The voltage applied was 30, 35, 38, and 40 V. (**c**) The current under a stress of *V*_DS_ = 30 V shows an extremely high current remaining ratio of approximately 90% after more than 24 h of testing.

The contributions of the electrons and lateral electric field were studied relative to the gate and drain voltage control when HVDS was applied, respectively. Figure 4a shows the characteristic lifetime vs. the leakage current between source and drain depending on the gate voltage (0, 2, 5, 10 V). The source and drain leakage current changed by two orders of magnitude from 10^−7^ A (@ V_g_ = 0 V) to 10^−5^ A (@ V_g_ = +10 V), which includes the leakage level when HVDS was applied. This indicates a strong relationship between the characteristic lifetime and the source and drain leakage current, and the relationship is shown along with the power law on the log-log plot. The reduction in the drain pulse level from 40 V to 35 V resulted in a gradual increase in the characteristic life (Fig. 4b). However, when the electric field was reduced to 30 V, almost no degradation was observed and an extremely high characteristic lifetime value was obtained. It is likely that the critical threshold driving voltage and current capability under the low level electric field (V_ds_ = 30 V, 3.75 × 10^4^ V/cm) would exhibit very little degradation if observed at this point of the test (Fig. 4c).

### Current degradation caused by radical changes in the input energy

**Figure 5:**
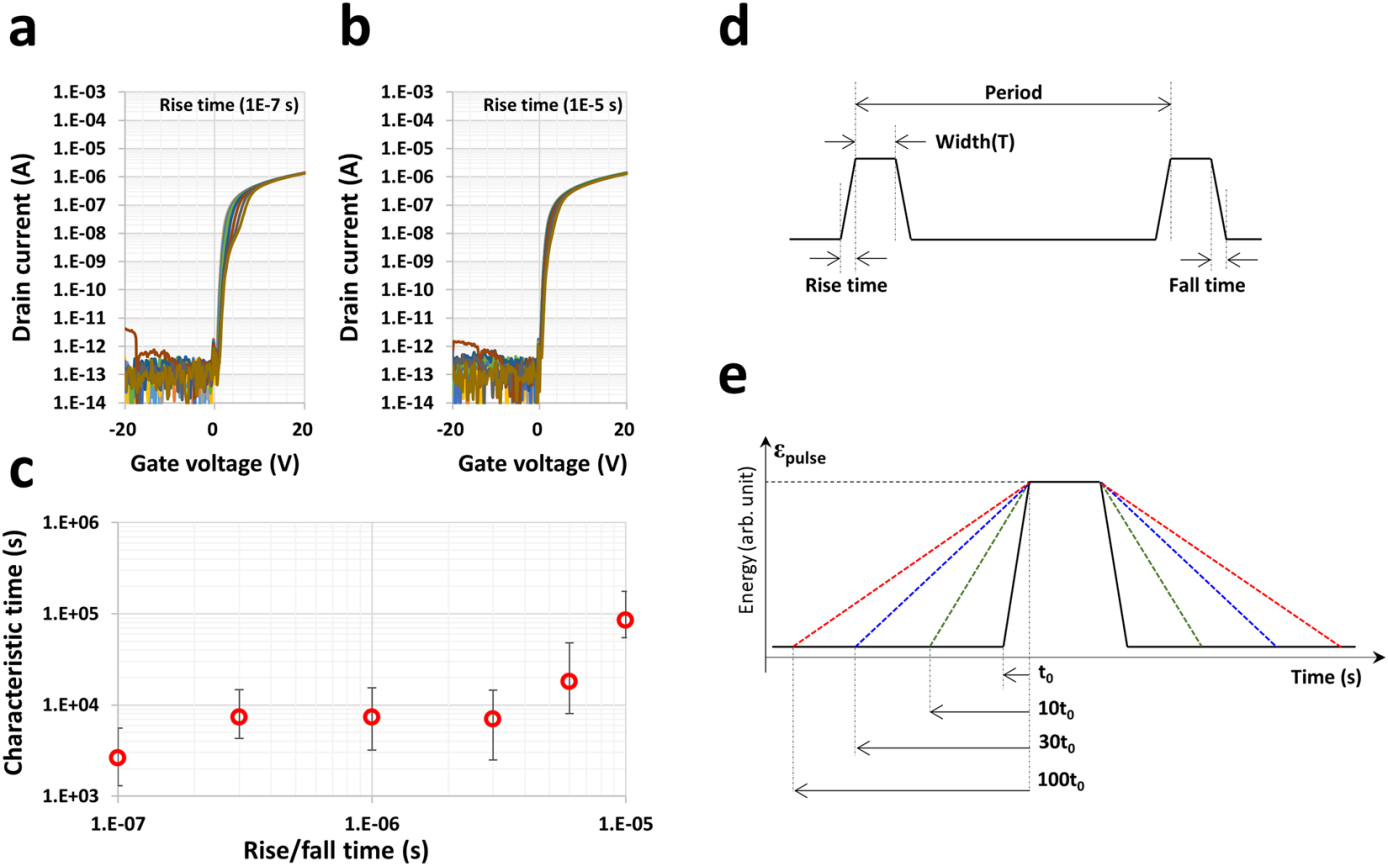
Device characteristics and schematic pulse diagram. (**a**) I_d_ − V_g_ characteristics at V_ds_ = 0.1 V for a short rise and fall time of 0.1 μs. The applied frequency and voltage were 1 kHz and 40 V at the drain (source is at zero voltage), respectively. (**b**) The change in the characteristics due to the reduced rise and fall time of 10 μs, which are compared with the results shown in **a**. The hump and current drop shown in **a** have either disappeared or been reduced. (**c**) The extracted characteristic times τ are proportional to the rise/fall times. (**d**) Schematic diagram defining the rise and fall times. (**e**) Schematic diagram of the pulse rise and fall times, t_0_, 10 t_0_, 30 t_0_, and 100 t_0_. Under the fixed pulse energy (stress voltage), the rising (or falling) time gives rise to the change in the energy slop, which is the instantaneous power.

In the HVDS test, the device not only exhibited enhanced current capability under low voltage stress, but also confirmed minimal drain side degradation under low frequency drive conditions, and no degradation under DC stress. This means that the leakage current had a limited effect on the activated hot electrons at the channel edge, whereas it showed a strong dependency on the degradation of the current capability, as shown in Fig. 4a. One of the key parameters is “the instantaneous power of the hot electron” which is the transient property on the electron working under the electric field variation. Under the fixed main parameters, including the frequency ( = number of stress times), vertical/lateral electric field, and leakage current between the source and drain, the pulse rise and fall times are tuned over two orders of magnitude when the pulse is introduced on the drain side (Fig. 5a and b). At a fixed frequency of 1 kHz, the characteristic time exponentially and non-monotonically increased as the pulse rise/fall time increased (Fig. 5c). Figure 5d shows a schematic illustrating the pulse timing and definition. The same pulse stress energy (ε_pulse_) was applied under equivalent conditions (e.g., pulse width, duty cycle, periodic time). The pulse energy (ε_pulse_) is:

2 $${\varepsilon }_{pulse}={\int }_{0}^{t0}p(t)dt,\,(for\,the\,pulse\,signal)$$

where *t* is the timing of the pulse signal, and *p*(*t*) is the instantaneous power as expressed in the slope of the rise (or fall) in the pulse energy vs. timing graph in Fig. 5e. This instantaneous power indicates the applied energy per unit time and increases as the time decreases under the fixed pulse energy, ε_pulse_. The hot electrons generated by the HVDS effectively transfer the energy to the bond states of the host structure, which causes some bonds to break and create dangling bonds.

### Defect generation near the conduction band

**Figure 6:**
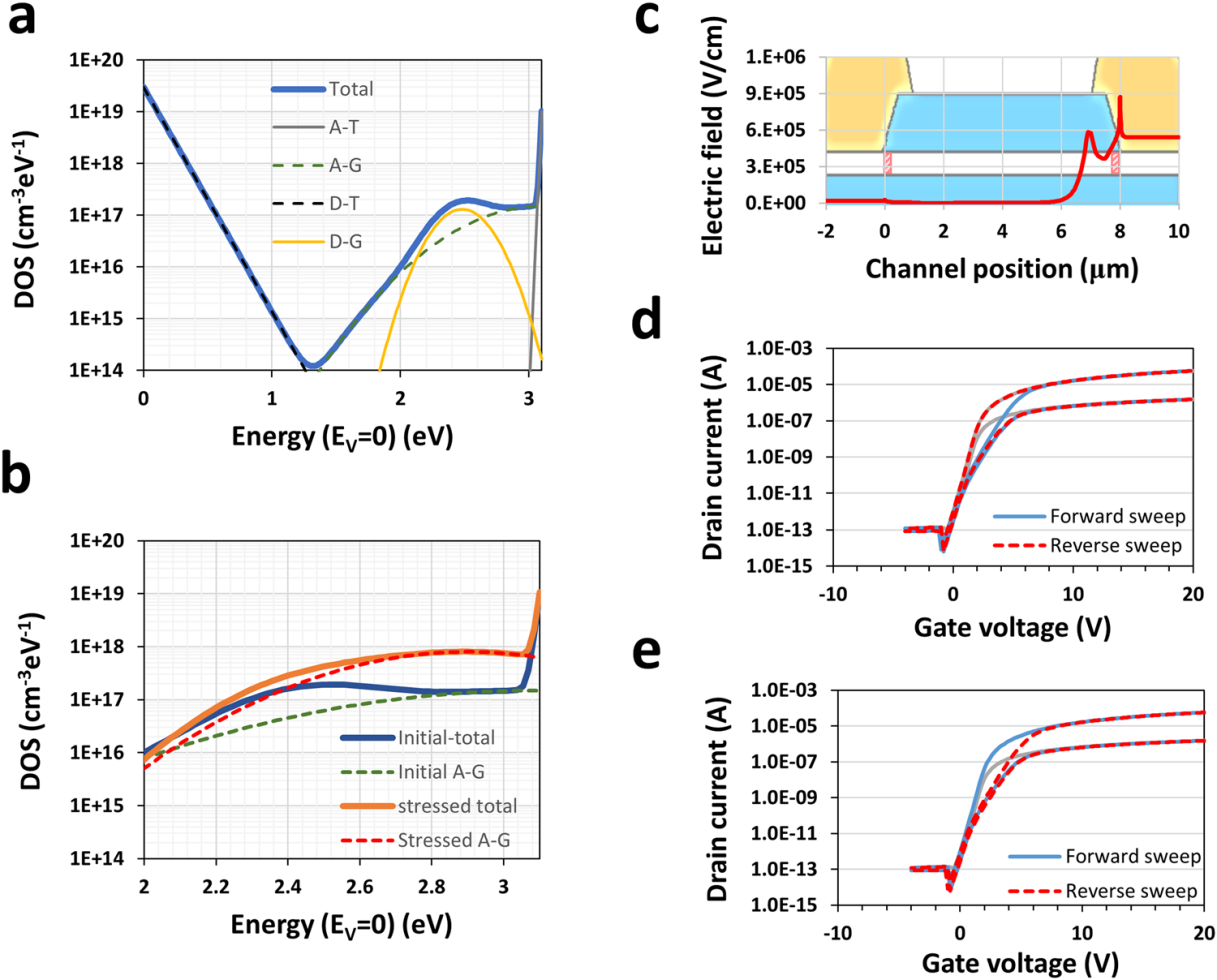
Local electric field and sub-gap state simulations. (**a**) Sub-gap states in the a-IGZO semiconductor. A-T, A-G, D-T, and D-G refers to the acceptor-like tail state, acceptor-like Gaussian state, donor-like tail state, and donor-like Gaussian state, respectively. (**b**) Local sub-gap density of states in the degradation area. Initial density of state on the nearby CBM is compared with the states after stress. (**a**) Distribution of electric field contours on the a-IGZO and gate insulator. (**c**) Electric field distribution as a function of the lateral distance (channel direction) at a 1 nm vertical depth from the top interface on the schematic cross sectional image of the device structure. There are two different peaks in terms of the etch stopper and back channel etch TFT structure. (**d**) Simulated I_d_ − V_g_ characteristics in terms of the defect (shown in **a**) positioned by 0.5 μm (near source electrode). Forward I_d_ − V_g_ sweep shows the current drop in the threshold region, whereas the reverse sweep shows an overlap with initial I_d_ − V_g_ characteristic (grey solid line). (**e**) Simulated transport characteristics in the case of generated defects near the drain electrode, 7.5 μm. The I_d_ − V_g_ characteristics show the opposite behaviour to those shown in (**d**), and trends similar to those in the measured I_d_ − V_g_, as shown in Fig. 2b.

To understand the impact and influence of the HVDS, the electric field profile was determined by a technology computer aided design (TCAD) simulation. The density of the local gap states (DOS) was determined such that the I_d_ − V_g_ properties were reproduced^21^. Figure 6a shows the initial band gap states along with the tailing and sub-gap states of the a-IGZO. The peak density of the donor-like tailing state (D-T) near the valence band and the donor-like Gaussian state (D-G) below the conduction band were calculated to be 3.0 × 10^19^ cm^−3^eV^−1^ and 1.3 × 10^17^ cm^−3^eV^−1^, respectively. The broad acceptor-like Gaussian state (A-G) of the initial state (without stress) of a-IGZO was positioned at the conduction band minimum (CBM) and its peak density was estimated 1.5 × 10^17^ cm^−3^eV^−1^. These sub-gap states were likely related to the oxygen environment in the a-IGZO, wherein one of the well-known states is “oxygen vacancies” in the crystal structure^22^. In the amorphous phase, the local Madelung (electrostatic) potential of the oxygen atoms is spread out on the energy states of the host matrix, which can be compared to that for the layered crystalline state^23^. The broad acceptor-like (Gaussian) state, which is one of these wide distributions, exhibits high densities in as-deposited IGZO films, but annealing at 200 °C or above decreases the density of the acceptor-like state^18^. Since annealing at the around 200 °C does not change the mass density, it would not cause a large displacement of the atomic structure with changes in the mass density, but only a small displacement of the atomic structure and/or the electronic states. A density increase of the acceptor-like Gaussian state, as shown in Fig. 6b, is able to reproduce the transfer curves degraded by HVDS. The degradation was reversed after annealing at 200 °C. This result suggests the possibility that the hot electrons under HVDS transfer energy via scattering with the a-IGZO system, which then forms high-density acceptor-like states. We confirmed that the magnitude of the local electric field near the drain electrode exponentially increased and the peak value of the electric field was observed at the edge of the drain. There are two electric field positions, and the second peak matched the end of the drain overlap position with the silicon oxide etch stopper layer on the active layer (Fig. 6c). The I_d_ − V_g_ characteristics were simulated as dependent on the local defects at three different positions on the channel: near the source (0.5 μm), the centre of the channel (4 μm), and near the drain (7.8 μm), which are marked on the a-IGZO channel. Figure 6d and e show the simulated I_d_ − V_g_ characteristics in the presence of local defects at positions of 0.5 and 7.8 μm, respectively. In the case of the defect positioned close to the source electrode, we verified the current drop in the forward I_d_ − V_g_ sweep. However, the current drop in the reverse sweep was clearly observed instead of the forward I_d_ − V_g_ sweep, which is consistent with the experiment results shown in Fig. 2b.

### Transient current effect

**Figure 7:**
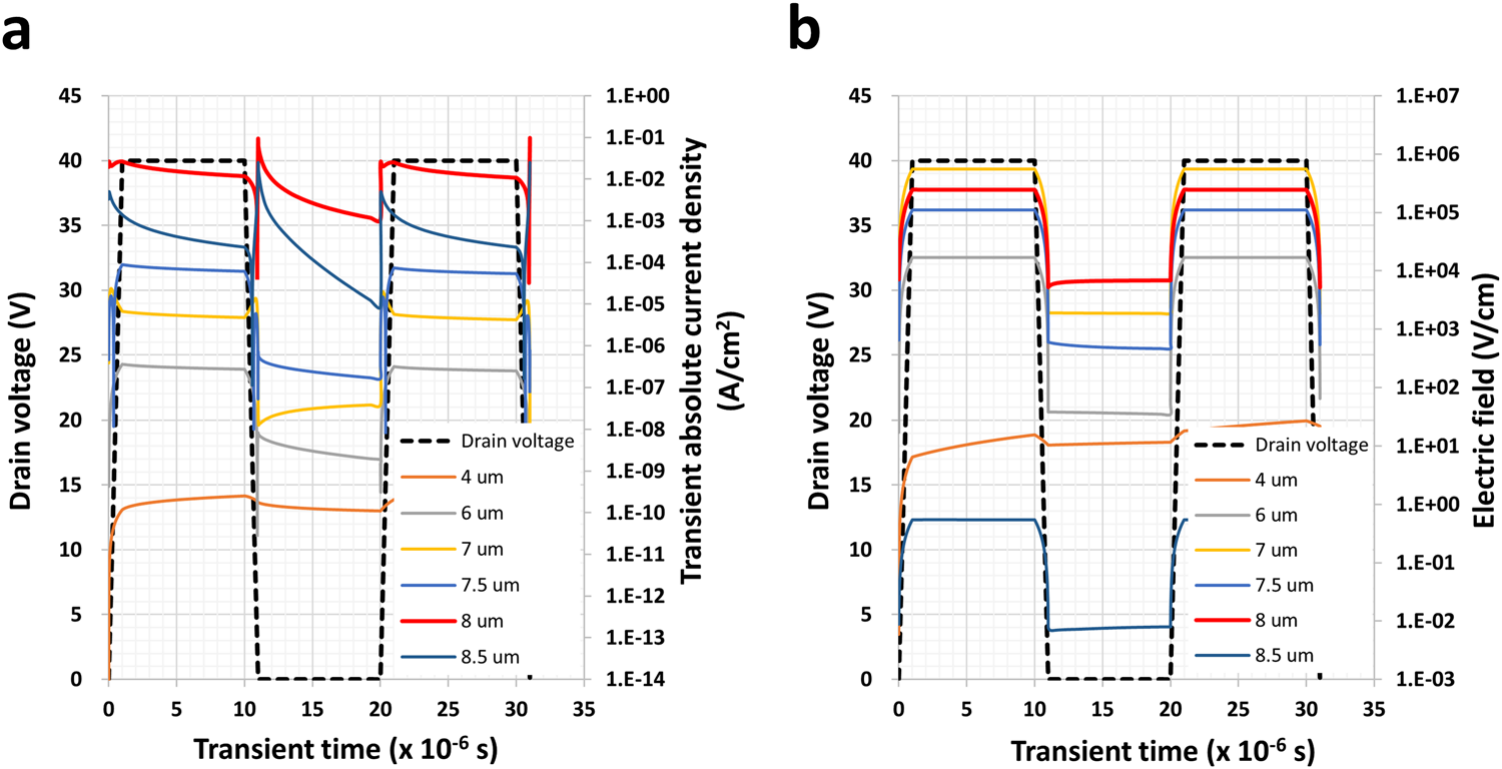
Transient current simulations. (**a**) Transient drain absolute current density distribution based on locations 4, 6, 7, 7.5, 8, and 8.5 μm from the edge of the source electrode in the Fig. 6c when the drain voltage is repetitively applied 0 V and 40 V. The 4 μm is the middle of channel length and, 8 μm is the location of the edge of drain electrode as shown in the Fig. 6c (8.5 μm is the location under the drain electrode). The transient current density is exponentially increased as close as the drain electrode. (**b**) The local electric field distribution is calculated by TCAD simulation.

It was confirmed that the AC pulse signal plays an important role in the reliability of the drive circuit, since no degradation was observed in the DC HVDS, even when equivalent tests were conducted. To understand the impact of the transient current density on the oxide semiconductor channel layer, the behaviour of the transient current density was determined by the TCAD simulation when the drain voltage was alternately increased and decreased. This transient current density on the drain was composed of two parts. The first is the gate coupling current caused by gate-drain overlap capacitance. The second is the current generated by the variation in the electron density in a-IGZO, which includes electrons emitted from the trap states. The local electric field accelerates the emission of electrons, and consequently, hot electrons form near the edges of the drain when the drain voltage is high. The results of the simulation showed that the current density and local electric field at the drain edge were higher than those at the centre of the channel or at the edge of the source. The transient current density at the drain edge (“position 8 μm”) fluctuated and spiked at the transient time around 10 μs when the drain voltage was first dropping toward zero, as shown in Fig. 7a. Whereas the transient current density increased and a small puffing out signal was observed when the voltage increased for the second time. The drain edge (“position 8 μm”) is the most likely area of degradation. The closer the drain, the higher the transient current density. Even the electric field at the edge of the overlap (“position 7 μm”) showed a higher value than that in the overlap area (“position 7.5 μm”) in Fig. 7b. The fluctuating and spiking current behaviour appears in the edge of the overlap area as it did at the drain edge. In the transient current simulation, the falling of the pulse signal causes more device degradation compared that caused by the rising effect, which is consistent with the results from silicon based devices.

## Discussion

**Figure 8:**
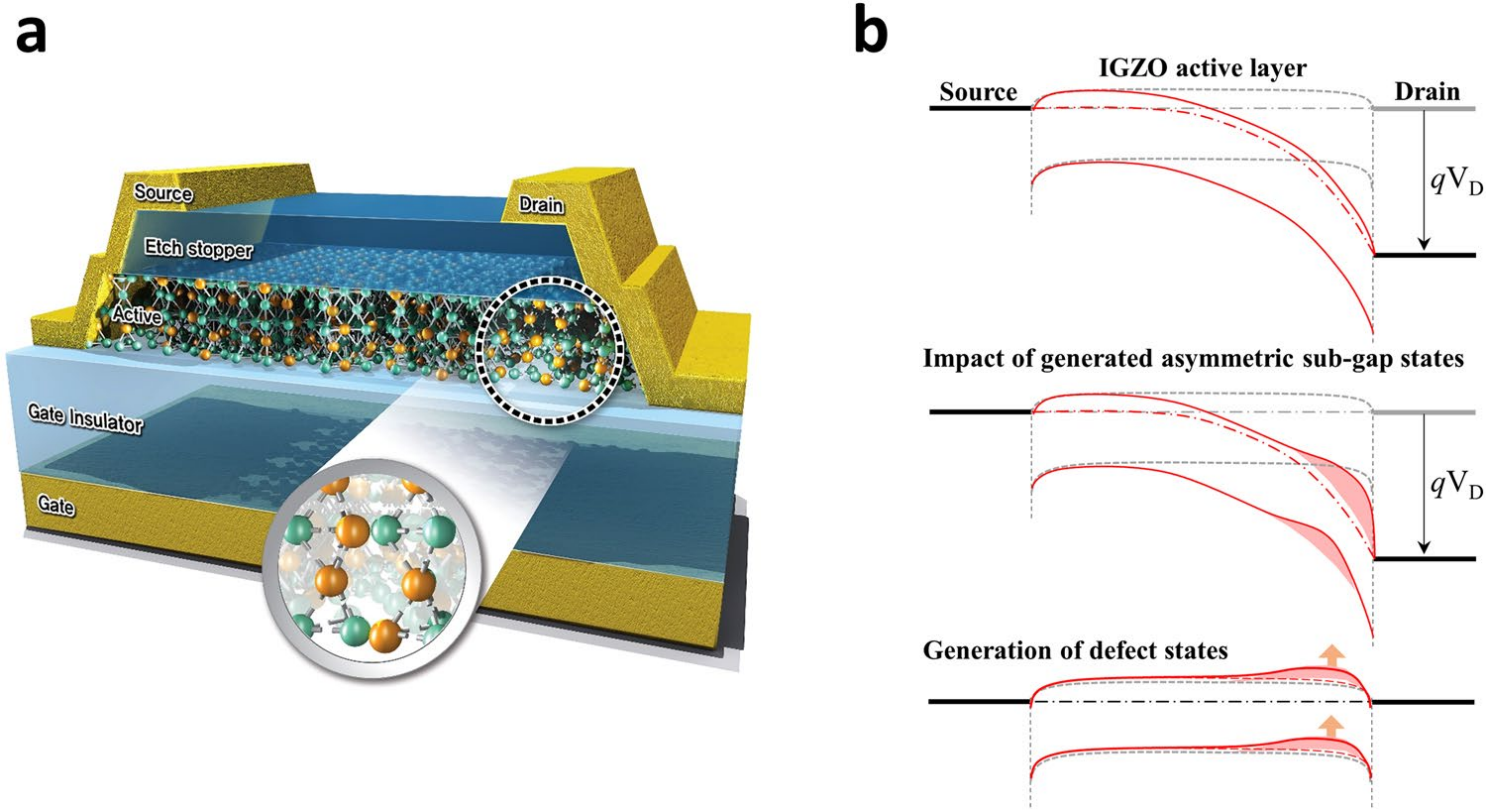
Schematic image and band state with defects in the degraded oxide TFT. (**a**) Three-dimensional schematic image of the degraded oxide semiconductor TFT by HVDS. At the near drain electrode, the disordered bonding has broken, which has created a dangling bond. The bond breakage (or unstable bonding with anti-sites) introduces a new obstacle to the flow of electrons. (**b**) The broken bonds create many defect states on the IGZO active layer at the drain side, and consequently impair the current capability of the distorted local host band structure.

In this paper, we reported on the degradation of the drive capability and the asymmetric degradation caused by hot electrons when driving pulse signals were applied between the source and drain in oxide semiconductor TFTs. There is a series of phenomena that arise when driving oxide semiconductor TFTs such that the trap states close to the CBM and the leakage current in the channel when the gate is in the off-state supply electrons that are transformed into hot electrons when subjected to a pulsed high electric field on the drain. The persistent hot electron emission from the trap states accelerates to generate (or grow) the acceptor-like Gaussian states. Within these sequences, we realised through the TCAD simulation that the transient and emission currents of hot electrons when the pulse signal was falling were generated by the instantaneous variation of the signal and are likely key parameters (Fig. 8a) in terms of the degradation caused by the high voltage drive of an oxide semiconductor. This impact on the oxide TFT creates defect states (Fig. 8b) at the active layer near the drain side (asymmetry) where the high driving voltage is applied, and then consequently acts as an obstacle, and disturbs the electric transfer of the information via electrons (or holes).

In a nutshell, the advantages of oxide semiconductor device technology, which includes IGZO, centres on its unique physical properties that enable the operation of power-efficient high resolution displays and oxide memory devices. However, even though these advantages are beneficial in electronic devices, there are disadvantages associated with the use of oxide semiconductors. For example, the material is a metal oxide bonded by ionic chains, and the high reactivity and mobility of oxygen may result in dangling bonds that can cause various defect states. In addition, the performance of oxide devices gradually drops when subjected to high frame rates due to the stress caused by the high voltage drive. The discovery and analysis process outlined in this paper should be applied to ensure the reliable design of integrated circuits that consist of oxide semiconductor devices, such as the gate driver circuits in display devices. The results of this study can be used to inform the design of oxide semiconductor devices and materials, such as a-IGZO, that are intended to be used as the active layer in TFTs.

## Methods

### Device Fabrication

A molybdenum (Mo) gate electrode (150 nm) was formed on an amorphous SiOx/Si substrate, and a 200 nm thick-SiOx single layer was then deposited at 380 °C by chemical vapor deposition (CVD) as the gate insulator (GI). An InGaZnO layer with a thickness of 40 nm was then deposited at 100 °C on the SiOx GI from a sintered IGZO ceramic target (Advanced Nano Products Co., Ltd.) by RF magnetron sputtering with a mixed gas ratio of Ar: O^2^ = 90: 10. A 100 nm thick etch stopping layer of SiOx was prepared by CVD on the active layer at 300 °C. The source and drain electrodes were formed using Mo of 150 nm. Finally, the fabricated IGZO TFTs (W/L = 40/8 um) were annealed in ambient air at 350 °C for 2 h before electrical measurements were conducted.

### TCAD Simulation

The simulation was conducted using the Atlas simulator package (Silvaco Co. Ltd.), and the following characteristic parameters were used as the input values. The relative permittivity of the silicon oxide as the gate insulator and a-IGZO as the channel semiconductor were 3.9 and 13, respectively. The electron affinity of a-IGZO was 4.1 eV. The band gap and mobility were 3.1 eV and 12 cm^2^V^−1^S^−2^, respectively. The effective density of the states in the conduction and valence bands were 5.0 × 10^18^ cm^−3^ and 4.6 × 10^19^ cm^−3^, respectively.

### Data Availability

The datasets generated during and/or analysed during the current study are available from the corresponding author on reasonable request.
